# Activation of nemo-like kinase in diamond blackfan anemia suppresses early erythropoiesis by preventing mitochondrial biogenesis

**DOI:** 10.1016/j.jbc.2024.107542

**Published:** 2024-07-09

**Authors:** Mark C. Wilkes, Aya Shibuya, Y. Lucy Liu, Kailen Mark, Jaqueline Mercado, Mallika Saxena, Ryan S. Sathianathen, Hye Na Kim, Bertil Glader, Paraic Kenny, Kathleen M. Sakamoto

**Affiliations:** 1 Division of Hematology/Oncology, Department of Pediatrics, Stanford University, Stanford, California, USA; 2 Kabara Cancer Research Institute, Gundersen Medical Foundation, La Crosse, Wisconsin, USA

**Keywords:** erythropoiesis, anemia, mitochondrial biogenesis, ribosomopathy, hematopoiesis, pediatric diseases

## Abstract

Diamond Blackfan Anemia (DBA) is a rare macrocytic red blood cell aplasia that usually presents within the first year of life. The vast majority of patients carry a mutation in one of approximately 20 genes that results in ribosomal insufficiency with the most significant clinical manifestations being anemia and a predisposition to cancers. Nemo-like Kinase (NLK) is hyperactivated in the erythroid progenitors of DBA patients and inhibition of this kinase improves erythropoiesis, but how NLK contributes to the pathogenesis of the disease is unknown. Here we report that activated NLK suppresses the critical upregulation of mitochondrial biogenesis required in early erythropoiesis. During normal erythropoiesis, mTORC1 facilitates the translational upregulation of Transcription factor A, mitochondrial (TFAM), and Prohibin 2 (PHB2) to increase mitochondrial biogenesis. In our models of DBA, active NLK phosphorylates the regulatory component of mTORC1, thereby suppressing mTORC1 activity and preventing mTORC1-mediated TFAM and PHB2 upregulation and subsequent mitochondrial biogenesis. Improvement of erythropoiesis that accompanies NLK inhibition is negated when TFAM and PHB2 upregulation is prevented. These data demonstrate that a significant contribution of NLK on the pathogenesis of DBA is through loss of mitochondrial biogenesis.

Diamond Blackfan Anemia (DBA) is an inherited bone marrow failure syndrome associated with severe anemia, an increased risk of developing cancers and congenital malformations. Most DBA patients carry genetic mutations affecting ribosomal protein genes. The disease is characterized by moderate to severe macrocytic anemia with hypoplastic bone marrow and reticulocytopenia (1). Evidence supports a decrease in uncommitted hematopoietic progenitors (2) with a more severe restriction in the earliest committed erythroid progenitors (3, 4, 5). Long-term bone marrow culture assays from DBA patients indicate defects in megakaryocytic and granulocytic progenitors (6, 7) and, in rare cases, progression to complete aplasia (8) can occur. Improving therapy for DBA is an urgent unmet clinical need as many patients are treated with frequent blood transfusions and iron chelation, chronic steroids, or hematopoietic stem cell transplantation (2, 9), all of which carry significant morbidity and poor quality of life.

Nemo-like kinase (NLK) is an evolutionarily conserved serine/threonine kinase belonging to the proline-directed protein kinase superfamily, which consists of mitogen-activated protein kinases and cyclin-dependent protein kinases (10). NLK contributes to cell proliferation, differentiation, apoptosis, and morphological changes during early embryogenesis and nervous system development and is involved in the pathogenesis of several human cancers (11, 12). Overexpression of NLK in colorectal, laryngeal, and non-small cell lung cancer, as well as osteosarcomas and neuroblastomas correlate with poor prognosis and more aggressive tumors. In contrast, NLK is significantly downregulated in breast cancer tissues, and re-expression of NLK results in c-Myb downregulation, reduced proliferation, and increased apoptosis in MCF7 breast cancer cells (13). NLK expression is also reduced in prostate metastasis, hepatocellular carcinoma, and glioblastoma with less favorable clinical outcomes, suggesting NLK can serve as an oncogene or tumor suppressor, depending on cell context. NLK kinase activity regulates a diverse array of signaling pathways, including the Wnt/β-catenin, Activin, IL-6, and Notch signaling pathways (11). Raptor, c-Myb (14), ATF5 (15), FoxO1 (16), Lef1 (17) and HDAC (18) are recognized NLK substrates in different cellular contexts.

NLK is chronically hyper-activated in the erythroid progenitors of DBA patients, and NLK inhibition improves erythropoiesis in *in vitro* ribosome insufficient DBA models (1, 14, 19, 20). In these models, NLK activation in erythroid progenitors has been demonstrated to increase c-Myb phosphorylation and degradation, as well as Raptor phosphorylation and reduced autophagy (14), but how NLK activation contributes to DBA pathogenesis has not been established. Determining signaling components downstream of NLK activation in DBA has the potential to illuminate novel therapeutic targets that improve the treatment of DBA patients.

Recent studies have characterized the importance of mitochondrial biogenesis in early erythropoiesis. In an unbiased study, Liu *et al.* identified TFAM and PHB2 are upregulated and essential for the increased mitochondrial biogenesis that occurs in red blood cell expansion (21). Both TFAM and PHB2 mRNA levels are unchanged but protein levels are upregulated, indicating an mTORC1-regulated increase in translation of these transcripts. Notably, the translational increase is not global but rather a specific subset of mTORC1 sensitive transcripts, particularly those containing 5′TOP (terminal oligopyrimidine) sequences in their 5′ untranslated region (5′UTR) (21). As NLK can suppress mTORC1 activity (22) and the mTORC1 stimulant leucine can improve erythropoiesis in DBA models (23, 24) we sought to determine if NLK impacts DBA pathogenesis through mTORC1 and mitochondrial biogenesis.

Here we present evidence that NLK-mediated phosphorylation of Raptor suppresses mTORC1 activity. Reduced mTORC1 activity decreases the translation efficiency of factors essential for the induction of mitochondrial biogenesis and increases the turnover of mitochondria through mitophagy in ribosome insufficient early erythroid progenitors. Subsequently, this reduction in mitochondrial output contributes to aberrant erythropoiesis in the pathogenesis of DBA.

## Results

### mTORC1 activity is reduced upon NLK activation in ribosome insufficient erythroid cells

**Figure 1:**
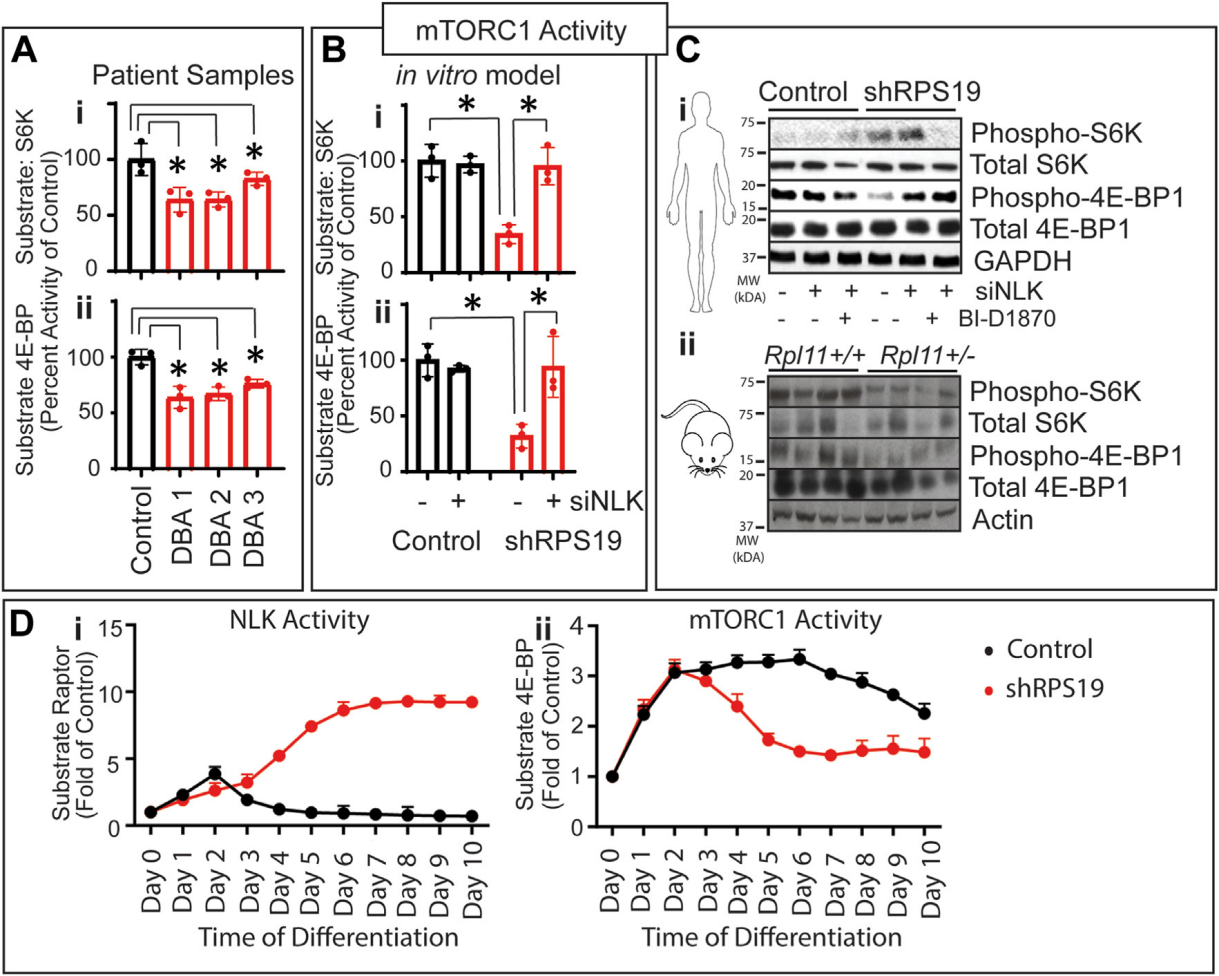
**NLK activation suppresses mTORC1 activity**. *A*, mononuclear cells from the bone marrow aspirates of one healthy donor and 3 DBA patients were differentiated in erythroid media for 5 days. Immunoprecipitated mTORC1 was subjected to *in vitro* kinase assay for phosphorylation of (i) S6K or (ii) 4E-BP1. *B*, cord blood CD34+ HSPCs were transduced with shRNA against RPS19 or control and non-targeting or siRNA against NLK and differentiated for 5 days in erythroid media and mTORC1 activity against (i) S6K and (ii) 4E-BP1 was assessed. *C*, (i) Human CD34+ HSPCs were transduced with shRNA against RPS19 or control and siRNA against NLK or NT. After 5 days of differentiation in the presence or absence of BI-D1870, cells were lysed and subjected to Western blot analysis. (ii) Sibling-matched *Rpl11*^+/Δ^ were fed tamoxifen or not for 14 days. CD71+ cells were isolated from blood and subjected to Western blot analysis. *D*, CD34+ HSPCs expressing control or shRPS19 were differentiated for the indicated days and (i) NLK and (ii) mTORC1 were immunoprecipitated. *In vitro* phosphorylation of Raptor by NLK, or 4E-BP1 by mTORC1, was analyzed. Data are represented as mean ± SD and significance is defined as *p* < 0.05 (n = 3). See also Fig. S1.

The mTORC1 complex contains the kinase mTOR and other proteins, including the regulatory protein Raptor. Phosphorylation of Raptor at S863 prevents mTORC1 from localizing to the outer lysosomal membrane where the complex is activated by Rheb (14, 22). In ribosome, insufficient erythroid progenitors, aberrantly activated NLK phosphorylates Raptor at S863 (14). We examined the impact of NLK-mediated phosphorylation of Raptor in mononuclear cells of bone marrow from three DBA patients and observed a statistically significant reduction in *in vitro* kinase activity of mTORC1 when compared to controls. Specifically, the *in vitro* phosphorylation of two well-characterized substrates of mTORC1, (i) S6K and (ii) 4E-BP1 were reduced by 36.1% (*p* = 0.0299), 35.9% (*p* = 0.0115) and 17.3% (*p* = 0.0277) and 36.1% (*p* = 0.0238), 32.9% (*p* = 0.0112), 23.8% (*p* = 0.0087) respectively, when compared to control (Fig. 1*A*).

Similarly, we assayed mTORC1 kinase activity in a common cell model of DBA, in which ribosome insufficiency is induced in hematopoietic progenitors using short hairpin RNA (shRNA) against ribosomal protein subunits mutated in DBA patients, such as RPS19. These cells display a similar erythroid differentiation failure as is observed in the bone marrow of DBA patients (14, 25, 26). As with the DBA patient cells, CD34+ hematopoietic stem and progenitor cells (HSPCs) were transduced with RPS19 shRNA, or control, and differentiated in erythropoiesis-promoting media for 5 days, showed reduced mTORC1 kinase activity. S6K phosphorylation (Fig. 1*B*i) was reduced by 65.7% (*p* = 0.0055) and 4E-BP1 phosphorylation was reduced by 68.1% (*p* = 0.008) compared to controls. Notably, mTORC1 kinase activity was rescued when siRNA against NLK was expressed in cells (S6K *p* = 0.0239, 4E-BP1 *p* = 0.0483) (Fig. 1*B*i and ii). As well, *Rpl11*^*+/Δ*^ mutant mice recapitulate the DBA phenotype (27). We analyzed mTORC1 activity in differentiated Lin^-^c-kit^+^ HSPCs from these Rpl11 insufficient mice and also observed reduced mTORC1 activity, which was predominantly restored when NLK is suppressed (Fig. S1).

Examination of the phosphorylation status of 4E-BP1 in ribosome insufficient erythroid progenitors correlated with the reduction of *in vitro* mTORC1 observed in Figure 1*B*. However, as reported previously (28, 29), S6K phosphorylation increases in ribosome insufficient cells. Unlike the phosphorylation of 4E-BP1, which is highly specific for mTORC1, S6K can be phosphorylated by other kinases, including RSK (30). As S6K phosphorylation was insensitive to NLK expression, we tested the impact of the non-specific RSK inhibitor, BI-D1870, on S6K and 4E-BP1 phosphorylation in RPS19 insufficient hematopoietic progenitors (Fig. 1*C*i) and *Rpl11*^*+/Δ*^ mutant mice (Fig. 1*C*ii). We found that BI-D1870 inhibits intracellular S6K phosphorylation, but had no impact on 4E-BP1 phosphorylation in the RPS19 insufficient hematopoietic progenitors (Fig. 1*C*i). As BI-D1870 does not target mTOR (31) this suggests that intracellular S6K phosphorylation in DBA is due to a different kinase and does not reflect mTORC1 status. In contrast, in the *Rpl11*^*+/Δ*^ mutant mice, we observed no increase in S6K or 4E-BP1 phosphorylation with BI-D1870 administration, but we did find that overall levels of phosphorylated S6K and 4E-BP1 (not total) are reduced in erythroid progenitors (Fig. 1*C*ii). Interestingly, total S6K (second panel) and 4E-BP1 (fourth panel) levels are variable across the mice, perhaps contributing to the variable penetrance of the disease in mouse models (27) (Fig. 1*C*ii). Finally, we examined NLK (i) and mTORC1 (ii) activity kinetics during erythroid differentiation in RPS19 insufficient hematopoietic progenitors and observed a strong induction of NLK activation around day 5, which correlated with a robust reduction in mTORC1 activity (Fig. 1*D*).

Our previously published data demonstrated that the aberrant activation of NLK in early erythroid progenitors in DBA results in phosphorylation of the mTORC1 complex on S863 of Raptor (14); from the data in Figure 1 we conclude that this phosphorylation of Raptor reduces the activity of mTORC1 leading to a decrease in phosphorylation of downstream substrates 4E-BP1 and S6K by mTORC1 in human and mouse models of DBA.

### NLK suppresses the translation of a subset of 5′TOP transcripts, including those required for mitochondrial biogenesis

**Figure 2:**
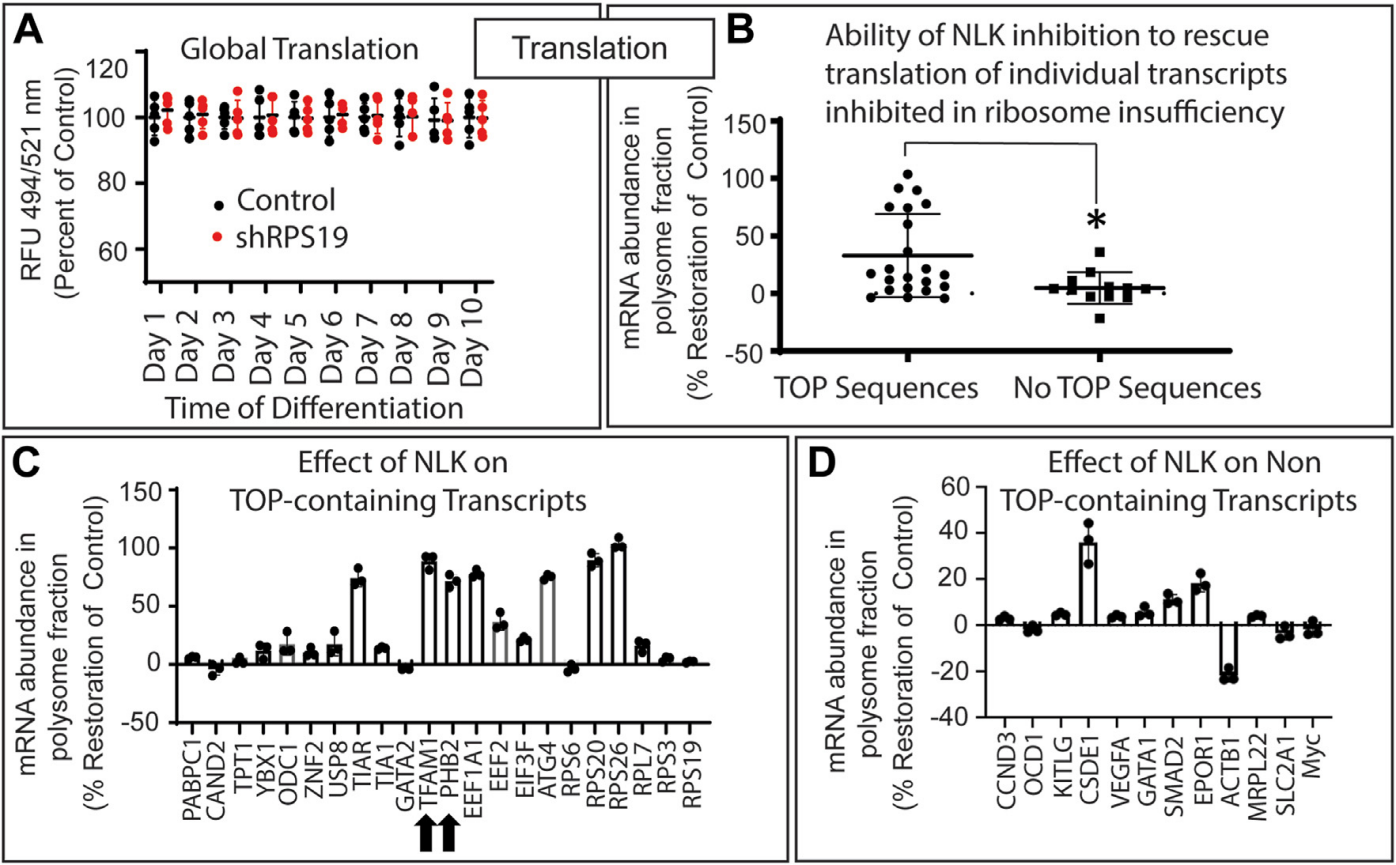
**NLK activation suppresses the translation of a subset of TOP-containing transcripts.***A*, differentiating HSPCs expressing control or RPS19 were incubated with OPP for 24 h at indicated times, and fluorescence at 494/521 nm was determined by flow cytometry. *B*, control or shRPS19 and non-targeting or siNLK expressing HSPCs were differentiated for 5 days. Cell lysates were separated by ultracentrifugation on a sucrose gradient and the abundance of 34 ribosome insufficiency-sensitive mRNAs sequences in polysome fractions were quantitated by qRT-PCR and normalized to total cellular fractions. To extrapolate the rescue effect, the variance between control and shRPS19 samples was calculated and designated 100%. Values from samples expressing both shRPS19 and siNLK were compared with 0% representing the same value as shRPS19 and 100% representing the same value as the control. Transcripts were segregated based on the possession of a TOP sequence in the 5′UTR or not and compared by student *t* test. The NLK translational rescue value of individual TOP-containing (*C*) and transcripts not containing a TOP sequence (*D*) are plotted. Three individual repeats were performed in triplicate. Data are represented as mean ± SD of the triplicate means and significance is defined as *p* < 0.05 (n = 3). See also Figs. S2 and S3.

One critical role of mTORC1 signaling is the regulation of protein translation. Active mTORC1 facilitates protein translation through the regulation of the initiation complex. The initiation complex binds mRNA transcripts, increasing their affinity to ribosomes to facilitate their recruitment and consequent translation efficacy. As was observed by others (25, 32) we did not detect a reduction in global translation (Fig. 2*A*) in ribosome insufficient cells. While mTORC1 stimulation has been demonstrated to increase all translation nonselectively, the transcripts most impacted by mTORC1 activity are those containing 5′Terminal Oligo Pyrimidine (5′TOP) motifs at the beginning (cap) of the 5′Untranslated region (5′UTR) (33, 34). This motif possesses a cysteine residue cap followed by 4 to 15 pyrimidines and a guanine/thymidine-rich sequence. Approximately one-third of transcripts possess a 5′TOP motif (33, 34).

As DBA patients have ribosome insufficiency, we postulated that the NLK-mediated reduction in mTORC1 activity would further reduce protein translation in DBA erythroid cells and that 5′TOP-containing transcripts would be particularly affected. We arbitrarily selected 22 5′TOP-containing transcripts and 12 non 5′TOP transcripts that all demonstrated at least a 2-fold reduction in translation efficiency in ribosome insufficient cells and determined the rescue effect of NLK inhibition on them (Fig. S2*A*). Examination of the abundance of these transcripts in polysome-containing fractions (actively being translated) revealed only seven of the 34 transcripts demonstrated a rescue greater than 50% upon NLK suppression (Fig. S2*B*). Notably, all seven transcripts contained 5′TOP cap sequences. Transcripts containing a 5′TOP were rescued by an average of 32.95% while transcripts without a 5′TOP were rescued by an average of 4.75% (*p* = 0.0015) (Fig. 2*B*). Although far from comprehensive, our data are consistent with NLK exerting a specific impact on a subset of 5′TOP sequences, and that much of the influence of ribosome insufficiency on translation is mediated by other mechanisms. A genome-wide study also reported a trend toward 5′TOP sequences being disproportionally dysregulated in DBA (25). To confirm translation efficiency correlated with protein expression, we performed a Western blot analysis. We examined the protein expression of two transcripts that were suppressed in ribosome insufficiency but rescued by NLK inhibition (TFAM and ATG4), two transcripts that were suppressed by ribosome insufficiency but not impacted by NLK status (RPS6 and GATA1) and two transcripts not impacted by ribosome insufficiency or NLK (P38 or NLK). Protein expression and translation efficiency are highly correlated for all transcripts (Fig. S3).

Within the subset of 5′TOP containing mRNAs that were strongly rescued by NLK inhibition were TFAM and PHB2, two critical regulators of mitochondrial biogenesis and erythropoiesis (21). TFAM was downregulated 4.1-fold (*p* = 0.001) in ribosome deficient cells but rescued 89.4% (*p* = 0.0032) by suppression of NLK. PHB2 is downregulated 6.3-fold (*p* = 0.0012) in ribosome insufficiency and rescued 63.1% (*p* = 0.0028) by NLK suppression (Fig. 2*C*). Transcription factor A, mitochondrial (TFAM) binds mitochondrial DNA (mtDNA) to regulate packaging, stability, and replication of the mitochondrial genome (35). Prohibin 2 (PHB2) functions within the nucleus, cytoplasm, and mitochondria. Of non-5′TOP sequences, NLK inhibition rescued CSDE1 by 36.1% but all other transcripts were rescued by less than 15% (Fig. 2*D*).

**Figure 3:**
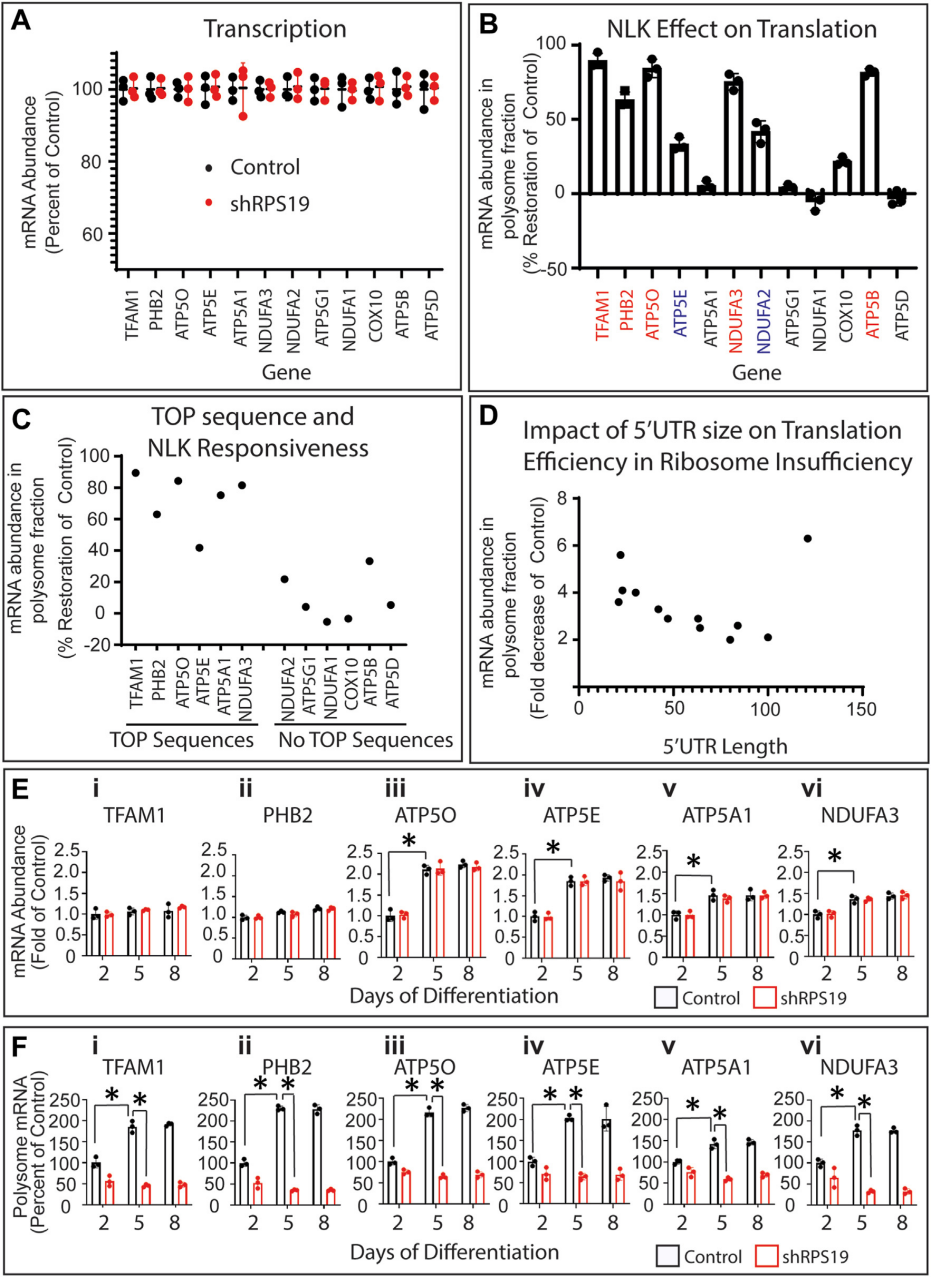
**NLK activation impacts translation of mitochondrial biogenesis transcripts that are upregulated during erythroid differentiation.***A*, CD34+ HSPCs were transduced with shRPS19 with or without siNLK and differentiated for 5 days. Total cellular mRNA for 12 mitochondrial genes that are impacted by ribosome insufficiency was assessed by qRT-PCR. *B*, after differentiation, mRNA expression in polysome fractions was assessed by qRT-PCR after sucrose gradient ultracentrifugation. The effect of NLK knockdown on transcript expression in RPS19 insufficient samples compared to controls was calculated. Transcripts in red are rescued by >50% and in blue by >20%. *C*, NLK rescue values for mitochondrial transcripts were segregated into those containing TOP sequences in the 5′UTR or not. *D*, the translation of mitochondrial transcripts in ribosome insufficiency was plotted again the nucleotide length of the 5′UTR. CD34+ HSPCs were transduced with control or shRPS19 and differentiated for 2, 5, or 8 days. Total cellular mRNA (*E*) and polysome-associated mRNA (*F*) were determined as above. Three individual repeats were performed in triplicate. Data are represented as mean ± SD of the triplicate means and significance is defined as *p* < 0.05 (n = 3). See also Figs. S4 and S5.

We next specifically examined how ribosome insufficiency and NLK inhibition impact the translation efficiency of nuclear genes that are involved in mitochondrial biogenesis (of note, mitochondrial genes are translated by specific mitochondrial ribosomes that are not impacted by DBA (36)). At day 5 of differentiation, we observed 12 mRNAs whose translation was reduced by at least 2-fold in RPS19 ribosome insufficient hematopoietic precursors including subunits of the ATP synthase complex, NADH: Ubiquinone Oxidoreductase complex, COX10, TFAM and PHB2 (Fig. S4*A*). No transcripts demonstrated differences at the mRNA level (Fig. 3*A*). Inhibition of NLK rescued the translation of five of these mRNAs (TFAM, PHB2, ATP5O, ATP5A1, and NDUFA3) by greater than 60% (shown in red) and an additional three mRNAs (ATP5E, NDUFA2, and ATP5B) by greater than 20% (shown in blue) (Fig. 3*B*). Six of the rescued mRNAs (TFAM, PHB2, ATP5O, ATP5E, ATP5A1, and NDUFA3) contain 5′TOP sequences and were rescued by NLK inhibition by an average of 72.6% (sd = 8.4%) (Fig. 3*C*). Six non 5′TOP-containing mRNAs were also rescued by NLK inhibition (NDUFA2, ATP5G1, NDUFA1, COX10, ATP5B and ATP5D) although this rescue was less than that observed for the 5′TOP containing mRNAs (Fig. 3*D*). Interestingly, all 12 DBA-impacted transcripts contained significantly shorter 5′UTR sequences (ranging from 21 to 121 nucleotides; see Fig. S4*B*); the average mRNA 5′UTR in humans is 210 nucleotides (37). Collectively, these data demonstrate that a select group of mRNA transcripts containing a 5′TOP sequence or shorter 5′UTR sequence and enriched for genes involved in mitochondrial biogenesis are predominantly impacted by ribosome insufficiency. Of these mRNAs, a subset is impacted by NLK activation, with those containing a 5′TOP sequence more likely to be affected.

As NLK activation and mTORC1 suppression in ribosome insufficient cells begins to peak at around day 5 in erythroid progenitors, we determined the total mRNA and ribosome-associated mRNA of the 6 NLK-responsive mRNA transcripts (TFAM, PHB2, ATP5O, ATP5E, ATP5A1 and NDUFA3) that are involved in mitochondrial biogenesis and dysregulated in RPS19 ribosome insufficient hematopoietic precursor cells at days 2, 5 and 8 of differentiation. Consistent with previous data, transcription of ATP5O (iii), ATP5E (iv), ATP5A1 (v) and NDUFA3 (vi) were moderately increased from day 2 to 5, but RPS19 mediated ribosome insufficiency had no impact. No significant increase in TFAM (i) (*p* = 0.294) or PHB2 (ii) (*p* = 0.5312) mRNA was observed (Fig. 3*E*). In contrast, polysome-bound mRNA analysis indicates significantly upregulated translation of all six transcripts from day 2 to day 5. TFAM (vii) translation increased 85.7% (*p* = 0.0087), PHB2 (viii) 230.5% (*p* = 0.0006), ATP5O (ix) 216.4% (*p* = 0.0026), ATP5E (x) 202.8% (*p* = 0.0015), ATP5A1 (xi) 42% (*p* = 0.0215) and NDUFA3 (xii) 77.6% (*p* = 0.008). Upregulation of these mRNAs was not observed in ribosome insufficiency (Fig. 3*F*). Consistent with this, Western blot analysis of TFAM, PHB2, ATP5E and ATP5A1 demonstrated that regulation of protein expression strongly correlates with translation when ribosome sufficiency and NLK expression are modulated in erythropoiesis (Fig. S5). These results confirm that in normal erythropoiesis there is an upregulation of critical factors involved in mitochondrial biogenesis in early erythropoiesis, and that the upregulation is not due to an increase in transcriptional output, but rather an increase in translation of these specific transcripts. In ribosomes, insufficient erythroid progenitors, upregulation of these critical regulators of mitochondrial biogenesis does not occur.

Taken together, our data demonstrate that while global translation efficiency is not reduced in RPS19-mediated ribosome insufficient erythroid progenitors, translation of a specific subet of mRNAs is significantly impacted. A subset of these downregulated mRNAs contain 5′TOP sequences; dysregulation of translation of these mRNAs is downstream of NLK-mediated suppression of mTORC1 and includes critical mitochondrial biogenesis factors such as TFAM and PHB2.

### Examination of mitochondrial biogenesis in ribosome insufficiency

**Figure 4:**
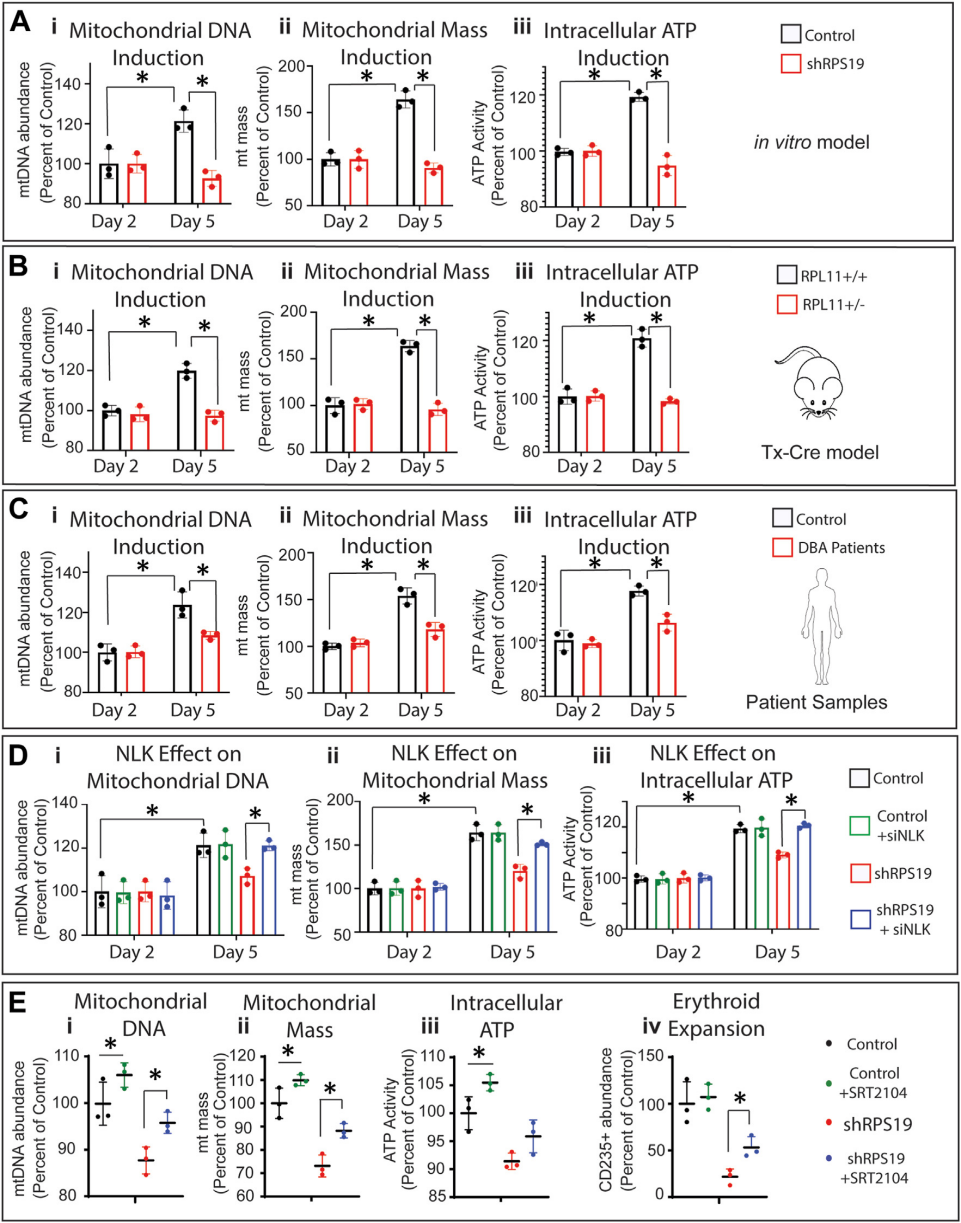
**Mitochondrial biogenesis is not upregulated in ribosome insufficiency due to NLK activity**. *A*, cord blood CD34+ HSPCs were transduced with shRPS19 or control and differentiated for two or 5 days in the presence of erythroid media. At indicated times, mtDNA was assessed by qRT-PCR (i), mitochondrial mass (ii) was assessed by flow cytometry analysis of mitotracker green uptake, and intracellular ATP (iii) was determined by CellTiter-Glo Luminescent Assay. *B*, Lin-Kit+ HSPCs from *Rpl11*^+/+^ or *Rpl11*^+/Δ^ mice were differentiated in erythroid media for two or 5 days and assessed for mtDNA, mitochondrial mass (ii) and intracellular ATP (iii). *C*, mononuclear cells from bone aspirates of a control and DBA patient were differentiated for 2 and 5 days prior to mtDNA (i), mitochondrial mass (ii) and intracellular ATP (iii) analysis. *D*, cord blood CD34+ HSPCs were transduced with shRNA against RPS19 or control and siRNA against NLK or non-targeting prior to differentiation for indicated times and assessment for mtDNA (i), mitochondrial mass (ii) and intracellular ATP (iii). *E*, human cord blood CD34+ HSPCs transduced with control or shRNA against RPS19 were differentiated in the presence or absence of SRT2104 and assessed for mtDNA (i), mitochondrial mass (ii) intracellular ATP (iii) and expansion of CD235+ erythrocytes (iv). Three individual repeats were performed in triplicate. Data are represented as mean ± SD of the triplicate means and significance is defined as *p* < 0.05 (n = 3). See also Figs. S6 and S7.

Upregulation of mitochondrial biogenesis is essential for erythropoiesis (21, 38) and is dependent on mTORC1 (21) and 4E-BP1 (39). We analyzed three measures of mitochondrial biogenesis in differentiating erythroid progenitors at day 2 and day 5 in RPS19-mediated ribosome insufficient erythroid progenitors or control cells (Fig. 4*A*). At day 5 mitochondrial biogenesis was significantly impacted in the RPS19 knockdown cells: (i) mtDNA was reduced by 23.6% (*p* = 0.0063); (ii) mitochondrial mass was reduced by 44.7% (*p* = 0.0017) and (iii) intracellular ATP was reduced by 20.6% (*p* = 0.007) when compared to controls. Unlike the RPS19 knockdown cells, the transduced controls showed a (i) 121.3% increase in mtDNA, (ii) 164.2% increase in mitochondrial mass, and (iii) 119.4% increase in intracellular ATP between day 2 and day 5. At day 2, no significant differences were observed in any measures of mitochondrial biogenesis between control and ribosome insufficient progenitors, indicating basal mitochondrial biogenesis is not impacted. We conclude that mitochondrial biogenesis in differentiating erythroid progenitors is significantly impacted at day 5 in RPS19-mediated ribosome insufficient erythroid progenitors.

We next looked at mitochondrial biogenesis in cells from *Rpl11*^*+/Δ*^ mutant mice, and observed suppression in ribosome insufficient cells but robust induction in differentiating control progenitors. In cells from *Rpl11*^*+/Δ*^ mutant mice mtDNA (i) was reduced by 2.5% at day 5 compared to an increase of 19.8% in controls (*p* = 0.0051), mitochondrial mass, (ii) was reduced by 4.0% compared to an induction of 63.7% in controls (*p* = 0.0031) and intracellular ATP decreased 1.7% compared to increasing 20.8% in controls (*p* = 0.0006) (Fig. 4*B*). Similarly, the induction of 23.6% of mtDNA (i), 54.1% mitochondrial mass (ii) and 17.7% intracellular ATP (iii) in controls, fell to 8.5% (*p* = 0.0056), 18.1% (*p* = 0.0143) and 6.4% (*p* = 0.0006) in cells differentiated from bone marrow aspirates of DBA patients (Fig. 4*C*). In agreement with previous studies indicating upregulation of mitochondrial biogenesis is a mTORC1-dependent process (21), the mTORC1 inhibitor torin prevented the induction of mitochondrial biogenesis at day 5 (Fig. S6).

To examine the role of NLK activation in mitochondrial biogenesis, we transduced siRNA against NLK in control or RPS19-mediated ribosome insufficient differentiating erythroid progenitors. MtDNA (i) was upregulated by 21.3% (*p* = 0.0225) in healthy controls and NLK suppression had no significant impact (*p* = 0.8907). In contrast, in RPS19-mediated ribosome insufficient erythroblasts, generation of mtDNA, which was only upregulated 7.2%, was robustly rescued by NLK suppression, increasing to 23.2% (*p* = 0.0245), representing a 109% rescue and approximating that observed in control cells. Further, the NLK suppression in RPS19-mediated ribosome insufficient erythroblasts also led to the restoration of control levels of mitochondrial mass (ii) 64.0% (*p* = 0.0065) and intracellular ATP (iii) to 20.6% (*p* = 0.0023) (Fig. 4*D*). Collectively, these data suggest that activation of NLK in DBA models is primarily responsible for preventing the upregulation of mitochondrial biogenesis in erythropoiesis.

As our data indicate, aberrant NLK activation impacts erythropoiesis by reducing mTORC1-driven mitochondrial biogenesis. We proposed that erythropoiesis could be improved in ribosome insufficient erythroid progenitors by stimulating mitochondrial biogenesis in a mTORC1-independent manner. The SIRT1 activating compound SRT-2104 stimulates mitochondrial biogenesis without impacting mitophagy (40). Stimulation of RPS19 ribosome-insufficient hematopoietic progenitors with SRT-2104 increased mtDNA (i), mitochondrial mass (ii), and intracellular ATP (iii) from 87.7% to 95.7% (*p* = 0.026), 73.1% to 88.2% (*p* = 0.0136) and 91.4% to 95.9% (*p* = 0.1191) of healthy controls at day 5 and improved erythroid (iv) expansion from 21.7% to 53.1% (*p* = 0.047) of controls at day 10. Mitochondrial biogenesis was also increased in controls treated with SRT-2104 but erythroid expansion did not significantly increase (*p* = 0.4615) (Fig. 4*E*). Ribosome-insufficient mouse hematopoietic progenitors treated with SRT-2104 also significantly increased mtDNA, mitochondrial mass, intracellular ATP, and erythropoiesis (Fig. S7). These data indicate that mitochondrial biogenesis is not optimal in ribosome insufficiency and stimulation of this process improves erythropoiesis.

**Figure 5:**
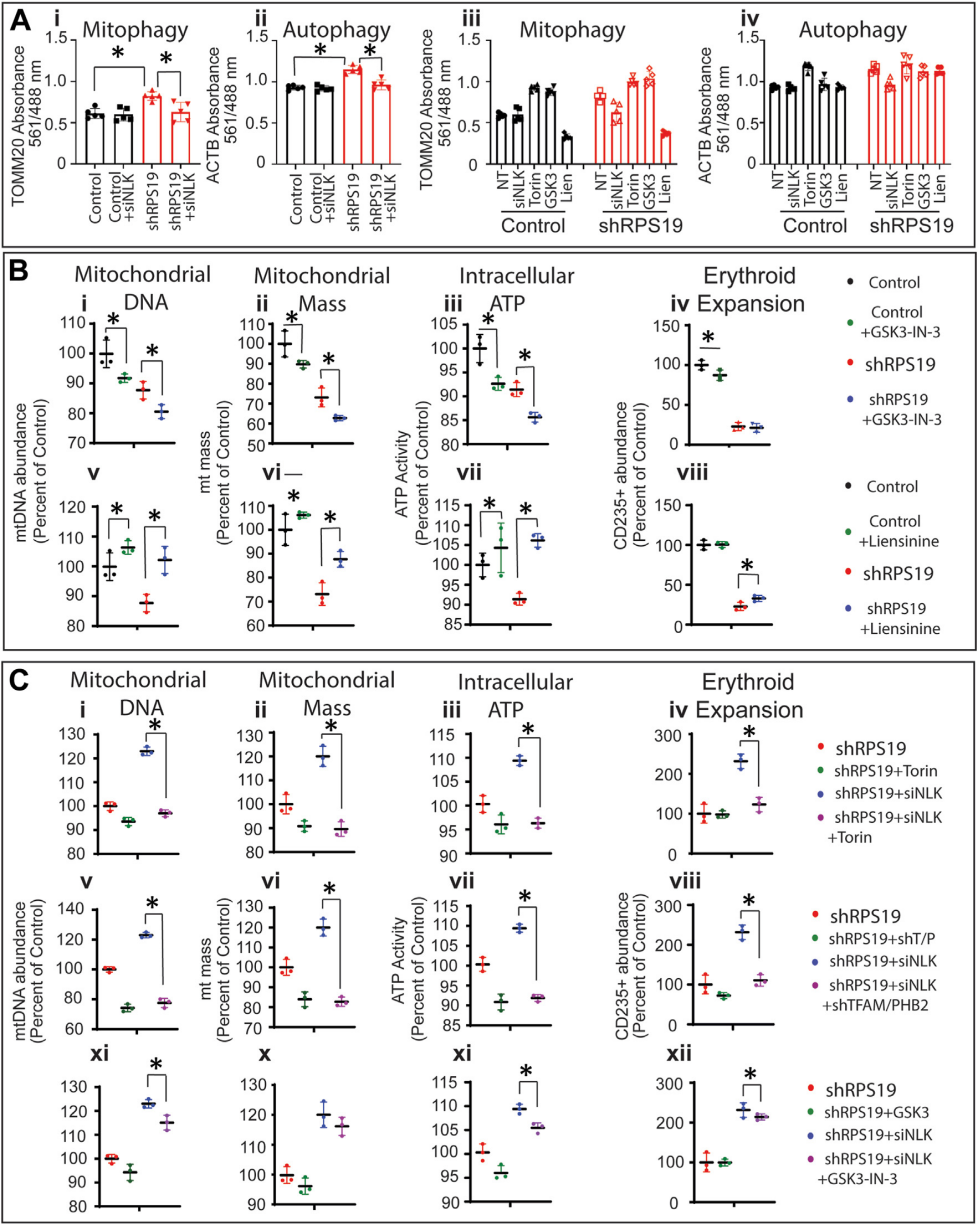
**Mitophagy cooperates with translation defects in suppression of mitochondrial output downstream of NLK and mTORC1 in ribosome insufficiency**. *A*, cord blood CD34+ HSPCs were transduced with control or shRNA against RPS19 with Keima-tagged TOMM20 (i) or ACTB (ii) alone, or TOMM20 (iii) and ACTB (iv), in the presence of torin, GSK3-IN-3, liensinine or siRNA against NLK. The ratio of free to lysosome-associated Keima was determined by comparing excitation at 488 and 561 nm respectively. *B*, cord blood CD34+ HSPCs transduced with control or shRNA against RPS19 were differentiated in the presence or absence of GSK3-IN-3 (*upper*), liensinine (*lower*), and assessed for mtDNA (*far left*), mitochondrial mass (*middle left*) intracellular ATP (*middle right*) and expansion of CD235+ erythrocytes (*far right*). *C*, cord blood CD34+ HSPCs were transduced with shRNA against RPS19 and either siRNA against NLK or control in the presence of Torin (*upper*), co-transduction with shRNA against TFAM and PHB2 (*middle*) or GSK3-IN-3 (*lower*). mtDNA (*far left*), mitochondrial mass (*middle left*), intracellular ATP (*middle right*), and expansion of CD235+ erythrocytes (*far right*) were assessed. Three individual repeats were performed in triplicate. Data are represented as mean ± SD of the triplicate means and significance is defined as *p* < 0.05 (n = 3 or 5). See also Fig. S8.

Decreased mitochondrial output can also occur because of an increase in mitochondrial turnover through mitophagy. The activity of mTORC1 regulates autophagy processes, including mitophagy (41) and NLK activation increases general autophagy in ribosome insufficiency (14). This prompted us to determine if dysregulation of mitophagy contributes to the reduced mitochondrial output in ribosome insufficiency. Keima is a fluorescent protein that changes excitation from 550 nm to 480 nm when the pH drop that accompanies trafficking from autophagosomes (pH7) to the lysosome (pH5) occurs. Keima is not degraded within the lysosome. Therefore the 550 nm/480 nm ratio indicates the ratio of free to lysosome-delivered Keima-tagged protein (42). By conjugating a Keima fluorescent tag to the mitochondrial protein TOMM20 we examined the flux between free and lysosome-contained TOMM20 (Fig. 5).

The ratio of TOMM20 (Ai) present in the lysosome significantly increased by 34.7% (*p* = 0.0008) in RPS19 ribosome insufficiency. Keima-tagged ACTB (ii) flux also significantly increased by 31.7% (*p* = 0.0006). These data suggest the observed increase in mitophagy in RPS19 ribosome insufficiency is due to an increase in general autophagy (*i.e.*, increased autophagosome generation), not specific targeting of mitochondria to the autophagosomes. This finding agrees with previous reports of increased autophagy in DBA (14, 29, 43). Notably, TOMM20 and ACTB turnover returned to baseline when NLK expression was suppressed. (Fig. 5*A*–*I* and ii). Suppression of NLK or treatment with the mTOR inhibitor torin increased flux of both TOMM20 (iii) and ACTB (iv) in both control and RPS19 ribosome insufficient cells, whereas the mitophagy-inducing agent GSK3-IN-3 and mitophagy inhibitor liensinine both specifically impacted TOMM20 and not the control protein actin (ACTB) (Fig. 5*A* – iii and iv). This supports the hypothesis that increased mitochondrial turnover in ribosome insufficiency is due to increased autophagy as a result of NLK-mediated suppression of mTORC1. Further support for the hypothesis that increased mitophagy is due to an increase in general autophagy comes from analysis of LC3 production (Fig. S8). Incorporation of the LC3-staining fluorescent dye increased in ribosome insufficiency by 2.2-fold (*p* = 0.0049) and was inhibited when siNLK was expressed (1.1-fold – *p* = 0.0069). The mTORC1 inhibitor torin, similarly increased autophagy in controls, (3.2-fold – *p* = 0.0011) while the mTORC1 stimulator leucine prevented autophagy in ribosome insufficiency (1.3-fold – *p* = 0.0148). Collectively, these data indicate NLK increases general autophagy through suppression of mTORC1.

Having determined increased autophagy in RPS19 ribosome insufficiency, we next examined the role of mitophagy in normal or RPS19 ribosome insufficient erythropoiesis. Differentiating HSPCs were cultured in the presence of the mitophagy-inducing agent GSK3-IN-3 and mitophagy inhibitor liensinine. In control cells, the induction of mitophagy significantly reduced mitochondrial biogenesis with mtDNA (i) reduced by 8.3% (*p* = 0.0094) mitochondrial mass (ii) reduced by 10.2% (*p* = 0.0033), intracellular ATP (iii) reduced by 7.4% (*p* = 0.0112) and erythropoiesis decreased (iv) by 12.7% (*p*= 0.0417) (Fig. 5*B* – upper). Similarly, statistically significant reductions in mitochondrial biogenesis were observed in RPS19 ribosome insufficient cells (*p* values of 0.0332, 0.0052, and 0.0131 for mtDNA (i), mitochondrial mass (ii) and intracellular ATP (iii) respectively) but no statistically significant reduction was observed in the suppressed erythroblasts (iv) (*p* = 0.6482) (Fig. 5*B* – upper). The opposite effect was observed when differentiating HSPCs were treated with the mitophagy inhibitor liensinine. While mitochondrial biogenesis was increased in healthy control progenitors, robust increases were observed in RPS19 ribosome insufficient cells with mtDNA (v), mitochondria mass (vi) and intracellular ATP (vii) increased by 16.5% (*p* = 0.0314), 19.9% (*p* = 0.0168) and 16.2% (*p* = 0.0045). Further, while liensinine did not significantly improve erythropoiesis (viii) in control cells (*p* = 0.8072), erythropoiesis was improved by 14.4% (*p* = 0.0427) in ribosome insufficiency (Fig. 5*B* – lower). Collectively this indicates the induction of mitophagy does reduce mitochondrial biogenesis and erythroid expansion in ribosome insufficiency, albeit modestly.

We next sought to determine the extent NLK-mediated suppression of mTORC1 contributes to mitochondrial biogenesis and erythroid failure in DBA. In cells treated with the mTOR inhibitor torin, the transduction of siRNA against NLK had no rescue effect on the three output measures of mitochondrial biogenesis or erythropoiesis failure. Treatment with torin prevented rescue by NLK suppression, with reductions from 123% to 97% (*p* = 0.001) in mtDNA (i), 120% to 89.6% (*p* = 0.0034) in mitochondrial mass (ii), 109% to 95.7% (*p* = 0.0014) in intracellular ATP (iii) and 231.6% to 123.1% (*p* = 0.0092) in erythropoiesis (iv) (Fig. 5*C* – upper). Indeed, torin suppresses mitochondrial biogenesis to a greater extent than siRNA against NLK in ribosome insufficiency, probably because NLK only reduces mTORC1 activity, whereas torin almost completely inhibits the kinase (44). These data confirm that NLK impacts mitochondrial biogenesis through suppression of mTORC1.

Although a number of mitochondrial biogenesis transcripts are influenced by NLK in ribosome insufficiency, TFAM, and PHB2 are especially important in erythropoiesis (21). In support of this role, the introduction of shRNAs against TFAM or PHB2 similarly prevented the rescue of mitochondrial biogenesis and erythroid expansion when NLK was suppressed. When expressed alone, NLK siRNA improved mtDNA (v), mitochondrial mass (vi), and intracellular ATP (vii) by 123%, 120%, and 109.1% of controls respectively. In the presence of TFAM/PHB2 shRNA, this was reduced to 77.5% (*p* = 0.0015), 82.7% (*p* = 0.0013), and 91.9% (*p* = 0.0009) of controls. Erythropoiesis was rescued by 231.6% when NLK was suppressed, but knockdown of TFAM and PHB2 reduced the effect of NLK suppression to only 110% of controls (*p* = 0.0046) (Fig. 5*C* – middle).

Induction of mitophagy with GSK3-IN-3 did not fully prevent the rescue effect mediated by NLK suppression but did reduce mtDNA (ix) rescue from 123% to 115.1% (*p* = 0.047), mitochondrial mass (x) rescue from 120% to 116.2% (*p* = 0.162) and rescue of intracellular cellular ATP (xi) from 109.1% to 105.5% (*p* = 0.0267). This correlated to a statistically significant reduction in erythropoiesis (xii) from 231.6% to 214.1% (*p* = 0.0012) (Fig. 5*C* – lower). Although modest compared to the effect of TFAM and PHB2 expression, the data suggest an upregulation of mitophagy in ribosome insufficiency also contributes to reduced mitochondrial biogenesis and anemia. Our data demonstrate that NLK-mediated suppression of mTORC1 reduces the translation efficiency of factors essential for the induction of mitochondrial biogenesis and increases the turnover of mitochondria through mitophagy in ribosome insufficient early erythroid progenitors.

## Discussion

**Figure 6:**
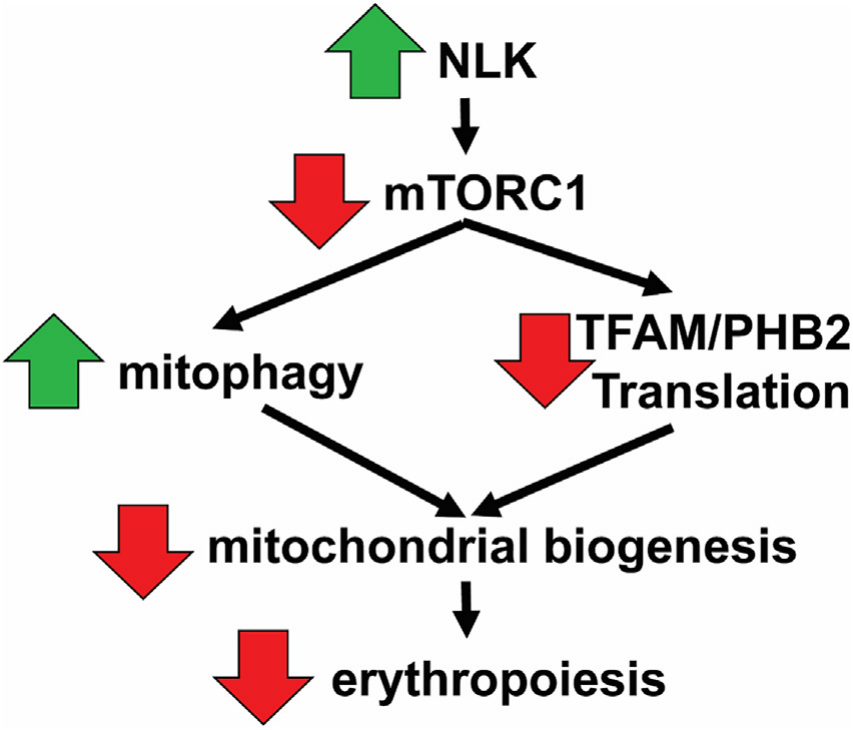
**Proposed model of how NLK activation in ribosome insufficiency contributes to failed erythropoiesis in DBA.** Proposed model of how NLK activation in ribosome insufficiency contributes to failed erythropoiesis in DBA. Activated NLK phosphorylates and suppresses mTORC1. The reduction in mTORC1 activity induces mitophagy and reduces translation of factors that induce mitochondrial biogenesis. Collectively, the mitochondrial output cannot be upregulated at day 5 and erythropoiesis is ineffective.

This is the first report describing the role of mitochondrial biogenesis, autophagy, and mitophagy in the pathogenesis of DBA. Accumulating evidence indicates that erythroid failure in DBA is not due to a global reduction in translation (25, 45). Instead, ribosome insufficiency appears to disproportionally affect transcripts that are translated less efficiently, such as GATA1, other ribosomal components (25) and as reported here, critical mitochondrial biogenesis factors. Examination of a subset of transcripts differentially downregulated in DBA revealed transcripts with a shorter 5′UTR tended to be less efficiently translated and transcripts containing a 5′TOP sequence were more likely to be influenced by NLK/mTORC1. Our data supports a model in which ribosome insufficiency leads to the activation of NLK. NLK phosphorylates and suppresses mTORC1 activity, which reduces 4E-BP1 phosphorylation to increase autophagy/mitophagy and reduce the translation efficiency of transcripts required for mitochondrial biogenesis. Around day 5, aberrant NLK activity begins to peak, a time frame that coincides with the upregulation of TFAM, PHB2, and other essential mitochondrial biogenesis factors during the differentiation of erythroid progenitors. In ribosome-insufficient cells, loss of these factors means mitochondrial biogenesis does not upregulate sufficiently, the impact of which is a failure of erythropoiesis (see Fig. 6).

Our data would suggest mitochondrial output was adequate to meet the energy requirements of differentiation and expansion in both control and ribosome insufficient progenitors prior to erythroid commitment. Mitochondrial biogenesis is robustly upregulated at about 5 days of differentiation (21) but the disruption in mitochondrial biogenesis in conditions of ribosome insufficiency was unexpected. However, considering the energy demands during the proliferative burst of the earliest stages of erythropoiesis when the DBA blockage is observed, a loss in mitochondrial output is consistent with the phenotype. This contrasts with earlier assumptions that erythroid failure in DBA is due to reduced global translation due to ribosomal insufficiency. Our data adds to accumulating evidence this is not the case, but rather that a reduction in the translation of a select set of transcripts that are translated with low efficiency inflicts the anemic phenotype. In DBA, erythroid differentiation disruption occurs at the earliest stage, prior to the enormous demands on the translation of hemoglobin accumulation and the dramatic upregulation of GATA1 that occurs in proerythroblasts. At this early stage, proliferation and the energy demands to fuel it are maximal, making these cells highly susceptible to disrupted mitochondrial biogenesis.

While we contend the disruption of the upregulation of mitochondrial biogenesis in early erythropoiesis is important in the DBA phenotype, it is only one of a number of deregulated processes that manifest at different stages of erythroid differentiation. The severity with which each of these processes is disrupted may contribute to the variability in severity, symptoms, and therapeutic responses of the DBA population. While increased mitophagy impacts basal mitochondrial output, the severe impact of ribosome insufficiency primarily occurs due to a failure to upregulate mitochondrial biogenesis coinciding with erythroid commitment – a process primarily driven by the translational upregulation of TFAM and PHB2 (21).

## Experimental procedures

### Cell culture

Primary human CD34+ hematopoietic stem and progenitor cells were purified from cord blood (New York Blood Center) by using magnetic-activated cell sorting (Miltenyi Biotec) and were cryopreserved. Upon thawing, cells were cultured in X-Vivo15 media (Lonza) containing 10% fetal bovine serum, fms-related tyrosine kinase 3 (50 ng/ml), thyroid peroxidase (50 ng/ml), interleukin-3 (IL-3; 20 ng/ml), interleukin-6 (IL-6; 20 ng/ml), and stem cell factor (50 ng/ml). At Day 3 10U/ml erythropoietin was added. HCoEpiC and BMSC stable cell lines expressing shRNA against RPS19 expressing RFP were generated by co-transfecting shRNA-carrying vectors (pLVTH) with neomycin-carrying vector (pcDNA3.1) using Lipofectamine 2000 (Thermo Fisher). Individual clones were harvested and expanded in 100 μg/ml neomycin and RPS19 expression was examined by Western blot and qRT-PCR. CD34+ progenitors were obtained from mononuclear bone marrow DBA patient samples using magnetic-activated cell sorting (Miltenyi Biotec) and differentiated for 12 days. Patient samples were obtained and provided by Dr Hanna Gazda and their collection and use were approved by the institutional review board at Boston Children’s Hospital.

Informed consent was obtained from affected individuals and their family members participating in the study.

### Lentiviral transductions

Primary CD34+ cells were transduced as published previously (14) using spinoculation (30 min at 800 g) at MOI 50 with lentivirus expressing shRNA against RPS19 or luciferase (Luc), siRNA against NLK or a non-targeting sequence. Virus co-expressed GFP, RFP, mCherry, or puromycin to enable selection. All assays were performed after FACS sorting at the indicated time of differentiation. Knockdown of TFAM and PHB2 was achieved by subcloning shTFAM and sh*PHB2* into the lentiviral vector prior to transduction Sequences are listed in Table S1.

### Compounds

SD208 (Tocris), metformin (SelleckChem), ginsenoside Rb1 (SelleckChem), SRT-2104 (SelleckChem), GSK3-IN-3 (MedChemExpress), liensinine (MedChemExpress), and Torin (SelleckChem), were diluted in dimethylsulfoxide (DMSO). Inhibitors were added to cells at indicated concentrations with a final DMSO concentration of 0.5%. EC_50_ calculations were obtained using 8 to 9 concentrations of compound and generated using the IC_50_ Calculator software by Prism.

### Flow cytometry

For cell surface flow cytometry, cells were incubated with human Fc receptor binding inhibitor (#14–9161−73; eBioscience) followed by primary antibodies CD235−APC (#306607; BioLegend) CD41-FITC (#303703; BioLegend) and CD11b-PE/Cy5 (#101209; BioLegend) or CD-71-APC (BD551374; BD Biosciences), or Ter119-FITC (#11-5921-82; Thermo fisher). Lysosomal flux measurements were obtained as described (42). The 561/488 nm ratio distribution graph was processed in Prism software after exporting the 488 and 561 nm ex./em. intensities of individual cells (10,000 cells) were exported. Data were collected on a DxP10 flow cytometer (Cytek) and analyzed by using FlowJo Software, v.9.7.2.

### Kinase assays

For NLK kinase analysis, cultures were treated as indicated and lysed for 30 min at 4 °C in 750 μl of kinase lysis buffer (50 mM Tris [pH 7.4], 5 mM EDTA, 250 mM NaCl, 0.1% Triton X-100, 50 mM NaF, 0.1 trypsin inhibitor unit of aprotinin per ml, 50 μg of phenylmethylsulfonyl fluoride per ml, 100 μM sodium vanadate, 1 μg of leupeptin per ml). Extracts were clarified, and equivalent protein was incubated overnight at 4 °C with antibody (NLK: #AB97642; Abcam). As mTOR activity is lost if dissociated from the complex, mTORC1 was extracted in a mild lysis buffer (50 mM Tris–HCl (pH 7.7), 10% (w/v) glycerol, 1% (w/v) Tween 20, 2.5 mM ascorbic acid, 50 mg/L soybean trypsin inhibitor, 50 mg/L phenylmethylsulfonyl fluoride, 5.0 mg/L leupeptin, 5.0 mg/L aprotinin, and a protease inhibitor cocktail set III). Immune complexes were collected with Catch and Release V2.0 Reversible Immunoprecipitation System and diluted in kinase buffer (25 mM Tris [pH 7.4], 10 mM MgCl2, 1 mM dithiothreitol). When cell numbers were limiting, NLK activity was amplified by adding 0.5 μg dephosphorylated NLK prior to performing kinase reaction. 50 μl of kinase buffer containing NLK sample and 5 μM ATP was incubated in the presence of biotinylated substrate (NLK, c-Myb, or Raptor for NLK and S6K and 4E-BP1 for mTORC1) immobilized on streptavidin-coated 96-well plates. The kinase reaction was allowed to proceed for 30 min at 37 °C before kinase was removed. Because the substrate S6K can be dephosphorylated by PP2A we added 500 nM Okadaic acid when assaying S6K phosphorylation. Phosphorylation of the substrate was detected by incubating for 30 min with a combination of phospho-Serine-HRP (ab9334; Abcam), phospho-Threonine-HRP (sc-5267; Santa Cruz Biotechnology) and SuperSignal West Pico Chemiluminescent Substrate (Thermo Scientific). Alternatively, ATP conversion to ADP was detected using ADP-Glo kinase assay (Promega), as per the manufacturer’s instructions. Signal was detected at 428 nm by Synergy H1 hybrid multi-mode microplate reader (BioTek). Prior to kinase analysis, NLK c-Myb, Raptor, S6K and 4E-BP1 were immunopurified by Catch and Release V2.0 Reversible Immunoprecipitation System and biotinylated as per manufacturer’s instructions (EZ Link NHS Biotin; Thermo Scientific) and immobilized on Pierce NeutrAvidin-coated 96-well plates (Thermo Scientific). Background phosphorylation was removed by 30 min incubation in the presence of 0.1 unit/ml calf intestinal phosphatase (New England Biolabs).

### qRT-PCR

RNA was isolated using RNeasy Plus Mini Kit (Qiagen). RNA was transcribed into cDNA by using the iScript cDNA Synthesis Kit (Bio-Rad). The quantitative RT-PCR (qRT-PCR) reaction was run with iQ SYBR Green MasterMix (Bio-Rad) using the CFX384 Touch Real-Time PCR Detection System (Bio-Rad). 7SL small cytoplasmic RNA was used as an internal control. miRNA was quantified using TaqMan Small RNA Assays (Applied Biosystems) as per manufacturer’s directions and normalized to snoRNA. Fold change of mRNA was calculated by using the comparative Ct method.

### Polysome profiling

Polysome profiling was performed as described with modifications (21). Briefly, cells were treated with 100 μg/ml cycloheximide (CHX) for 5 min, harvested, washed twice with PBS containing 100 μg/ml CHX, and centrifuged at 300*g* for 5 min at 4 °C. Cells were resuspended in 425 μl hypotonic buffer (5 mM Tris-HCl pH7.5, 2.5 mM MgCl2, 1.5 mM KCl, protease inhibitor cocktail) and transferred to a pre-chilled 1.5 ml tube. Then 5 μl of 10 mg/ml CHX, 1 μl of 1M DTT, and 100U RNase inhibitors were added to cells followed by vortexing for 5s. Then 25 ul of 10% Triton X-100 and 25 μl of 10% sodium deoxycholate were added to cells followed by vortexing for 5s. The supernatant were transferred to a pre-chilled 1.5 ml tube after centrifugation at 21,000*g* for 5 min at 4 °C. The same OD amount of lysates from each sample were loaded onto a 10 to 50% sucrose gradient, and centrifuged at 35,000 rpm for 2 h at 4 °C using SW40Ti rotor in a Beckman Coulter Optima L-80 ultracentrifuge with no brake. Samples were analyzed on the Bio-Rad BioLogic LP system and BioFrac fraction collector, and chased with 60% (w/v) sucrose with bromophenol blue at 1 ml/min. Data were analyzed using the Bio-Rad LP Data View software. Polysome fractions were collected at 0.5 ml/fraction.

### Global translation

Global Protein translation was analyzed through the incorporation of O-Propargyl-puromycin (OP-puro) using the Global Protein Sythesis Assay kit (ab273286) from Abcam. Cells were incubated with OPP for 24 h at indicated times during differentiation. Fluorescence was observed at 494/521 nm.

### Mitochondrial DNA, mass, and intracellular ATP

Mitochondrial DNA (mtDNA) was determined using qPCR to measure the ratio of mtDNA *versus* genomic DNA. The primer sequences are in Table S1. Mitochondrial mass was determined using flow cytometry. Briefly, 0.5∼1 × 106 cells were incubated with 100 nM MitoTracker Green (Invitrogen) and antibodies for cell surface markers for 30 min at 37 °C, and analyzed on FACS with 488 nm excitation. Intracellular ATP was determined using the CellTiter-Glo Luminescent Cell Viability Assay (Promega) following manufacturer's protocol.

### Western blotting

Antibodies against NLK (#AB97642; Abcam; 1:500), phospho-S6K T389 (#ab60948; Abcam; 1:1000), S6K (#ab9366; Abcam; 1:1000), phospho-4E-BP1 S65 (#9451; Cell Signaling; 1:500), 4E-BP1 (#ab2606; Abcam; 1:1000), GAPDH (#MAB374; Millipore; 1:10,000) and actin (#sc-8432; Santa Cruz Biotechnology; 1:2000) were used according to manufacturer’s instructions. The target proteins were analyzed by using SuperSignal West Pico Chemiluminescent Substrate for horseradish peroxidase (Thermo Scientific). Densitometry was performed using Image J software (http://rsb.info.nih.gov/ij/).

### Mice

Inducible *Rpl11* heterozygous deletion mice were fed a standard chow diet *ad libitum*. When indicated, the standard chow diet was replaced by a tamoxifen diet (Teklad, Harlan Laboratories) to induce activation of the CreERT2 transgene. All animals were maintained at the Spanish National Cancer Research Centre (CNIO) under specific pathogen-free conditions, in agreement with the recommendations of the Federation of European Laboratory Animal Science Association (FELASA). All animal procedures were evaluated and approved by the Ethical Committee of the Carlos III Health Institute, Madrid, Spain (#54-2013-v2).

### Statistics

*p* values for statistical significance were obtained by using a paired Student *t*-test. Significance was designated as *p* < 0.05. The data are representative of at least three independent experiments (*n* = 3). Each independent experiment was performed in triplicate and reported as the mean. Therefore, when calculating the mean and standard deviation of the combined three independent experiments, the value reported is the mean of the means from the three sets of triplicate values.

## Data availability

All data supporting this study are reported within this article. Raw data are available from the corresponding author upon reasonable request.

## Supporting information

This article contains supporting information.

## Conflict of interest

The authors declare that they have no conflicts of interest with the contents of this article.
