# Supplementary material for: Activation of nemo-like kinase in diamond blackfan anemia suppresses early erythropoiesis by preventing mitochondrial biogenesis
Source: J Biol Chem. 2024 Jul 9;300(8):107542. doi: 10.1016/j.jbc.2024.107542 (PMC11345392; doi:10.1016/j.jbc.2024.107542)
Supplement: Supporting information [file mmc1.docx]

**Supporting Information
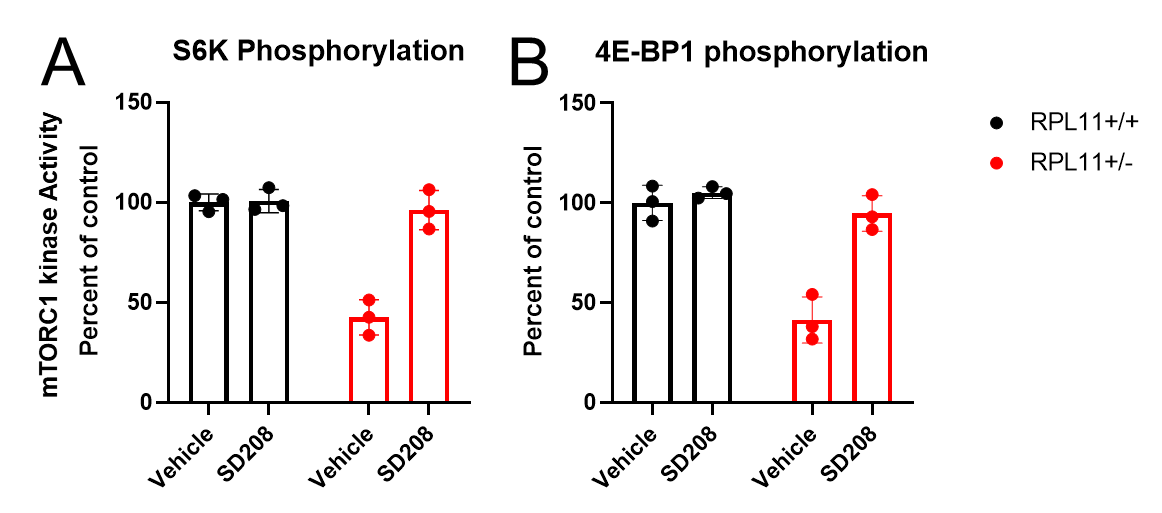
**

**Supplemental Figure 1**. mTORC1 activity is reduced in ribosome insufficient mouse HSPCs. Lin-Kit+ HSPCs from *Rpl11*^+/+^ or *Rpl11*^+/Δ^ mice were differentiated for 5 days and assessed for mTORC1 activity by in vitro kinase assay with S6K (A) and 4E-BP1 (B) as substrates. Three individual repeats were performed in triplicate. Data are represented as mean ± SD of the triplicate means and significance is defined as p<0.05 (n=3). Accompanies Figure 1.


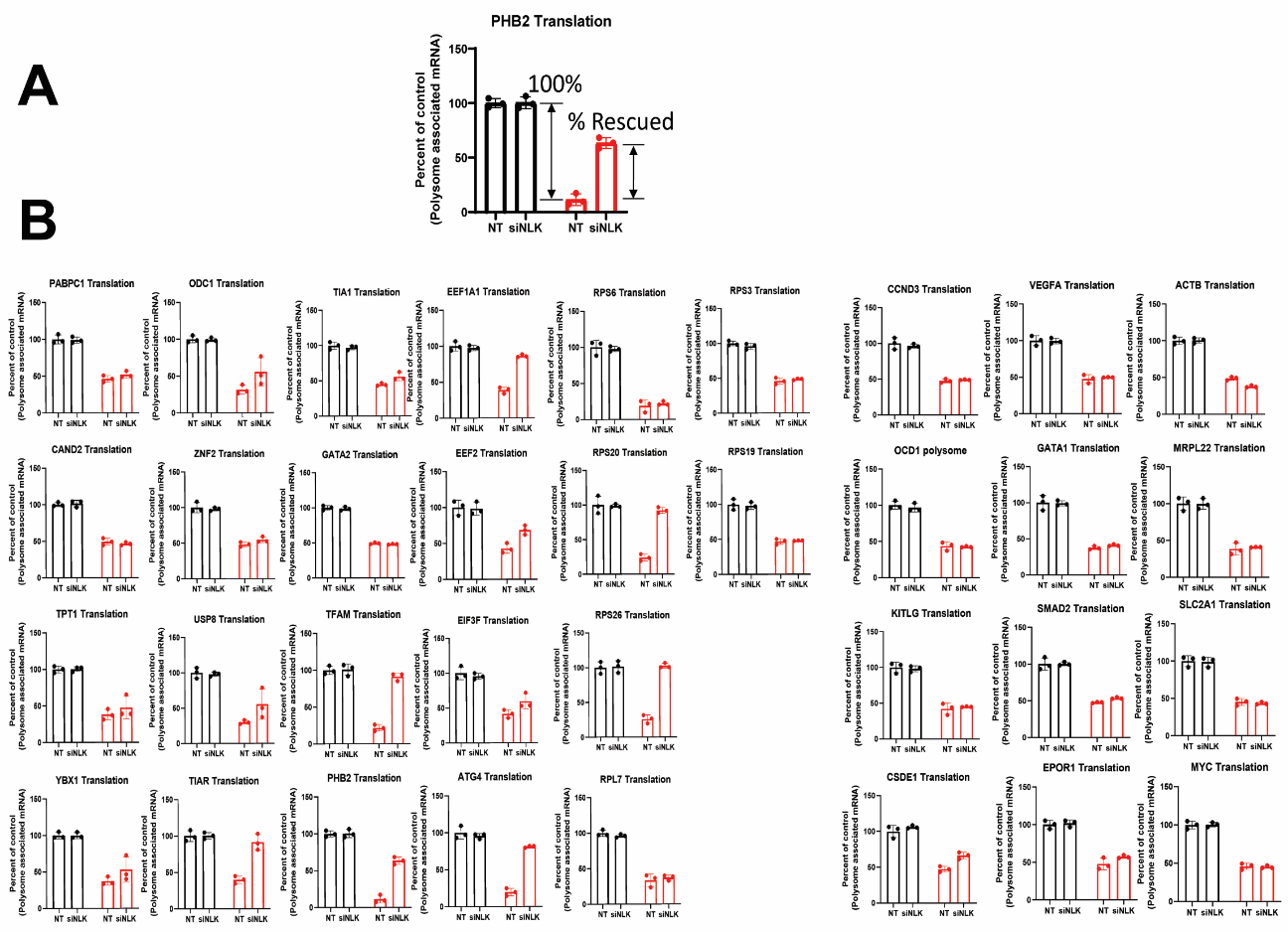


**Supplemental Figure 2**. Transcripts containing and not containing 5’TOP sequences in the 5’UTR are influenced by ribosome insufficiency and NLK. (A) Example of how the % rescued by NLK suppression is calculated. After normalizing to control, the difference between control and shRPS19 is calculated and assigned a value of 100%. Where the shRPS19/siNLK value falls on that scale is assigned the NLK rescue effect. (B) Control or shRPS19 and non-targeting or siNLK expressing HSPCs were differentiated for 5 days. Cell lysates were separated by ultracentifugation on a sucrose gradient and the abundance of 34 ribosome insufficiency-sensitive mRNAs sequences (minimum 2-fold reduction in ribosome insufficiency) in polysome fractions were quantitated by qRT-PCR and normalized to total cellular fractions. Three individual repeats were performed in triplicate. Data are represented as mean ± SD of the triplicate means and significance is defined as p<0.05 (n=3). Accompanies Figure 2.


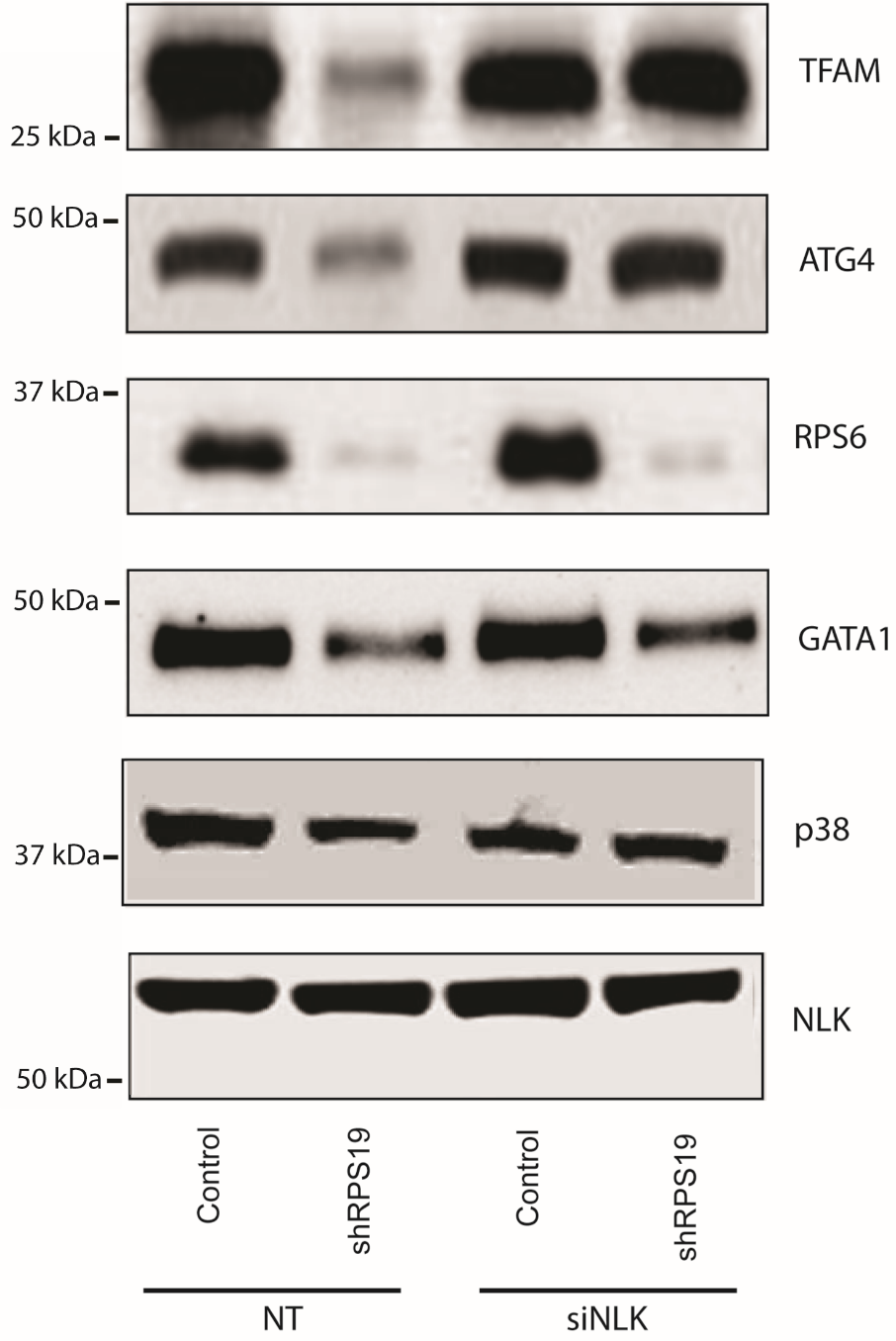


**Supplemental Figure 3**. Deregulated translation efficiency correlates with protein expression in ribosome insufficiency. Cord Blood CD34+ progenitors transduced with shRNA against luciferase (Control) or shRNA against RPS19 (shRPS19) in conjunction with siRNA against NLK (siNLK) or a non-targeting sequence (NT). Cells were differentiated for 5 days. Lysates from 4 identical assays were pooled to ensure sufficient protein. Samples were lysed and probed for TFAM, ATG4, RPS6, GATA, p38 and NLK protein expression by western blot. Accompanies Figure 2.

**
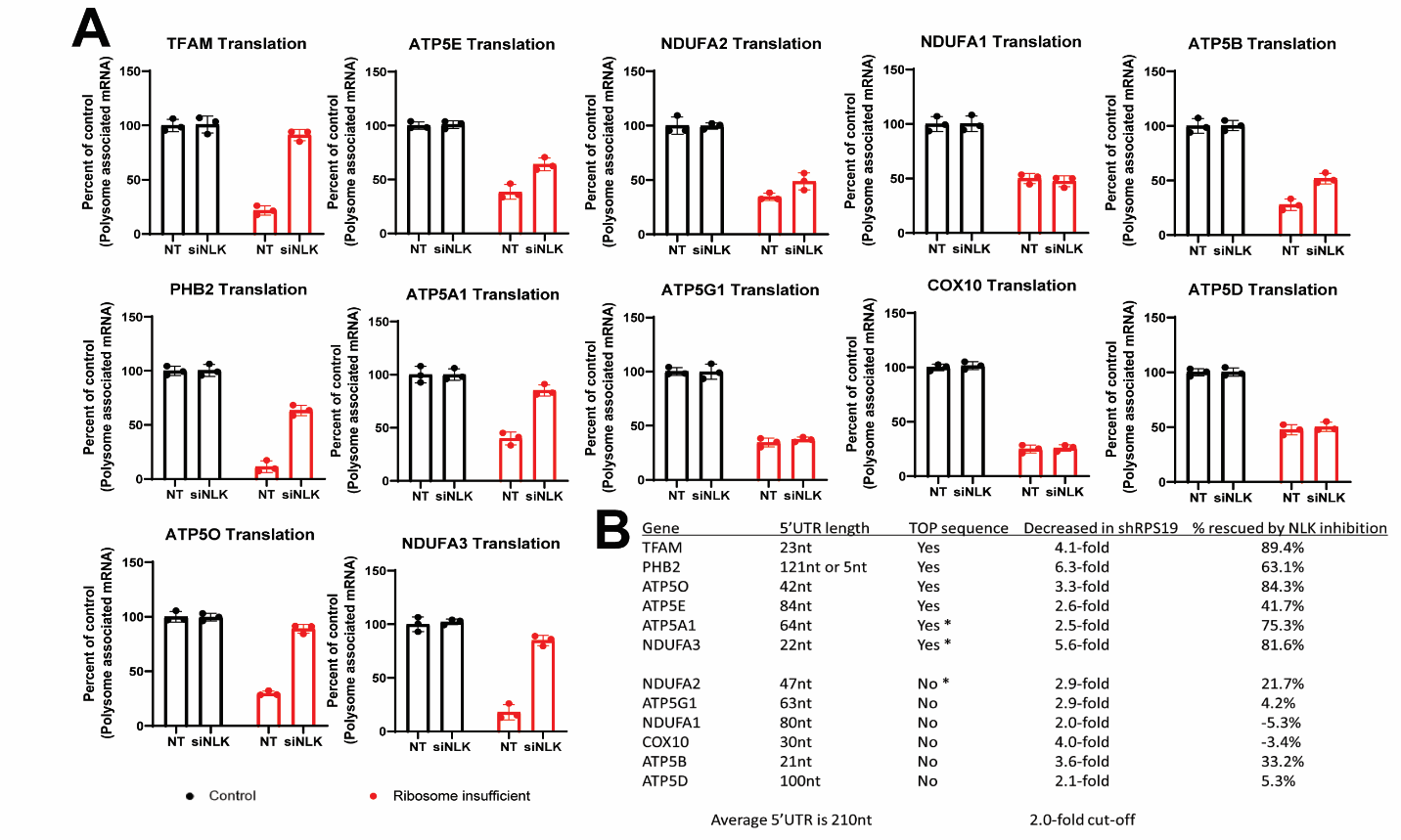
**

**Supplemental Figure 4**. Transcripts involved in mitochondrial biogenesis are disrupted in ribosome insufficiency in an NLK-dependent manner. (A) Control or shRPS19 and non-targeting or siNLK expressing HSPCs were differentiated for 5 days. The abundance of 12 ribosome insufficiency-sensitive mRNAs sequences (minimum 2-fold reduction in ribosome insufficiency) that are involved in mitochondrial function were quantitated from the polysome fraction after sucrose gradient ultracentrifugation by qRT-PCR. Values were normalized to total mRNA levels to account for differences in transcription. (B) The 5-UTR length, and presence or absence of a 5’TOP sequence is demonstrated, as well as the extent the transcript is influenced by ribosome insufficiency and the extent NLK contributes to the deregulation. Three individual repeats were performed in duplicate. Data are represented as mean ± SD of the duplicate means and significance is defined as p<0.05 (n=3). Accompanies Figure 3.

**
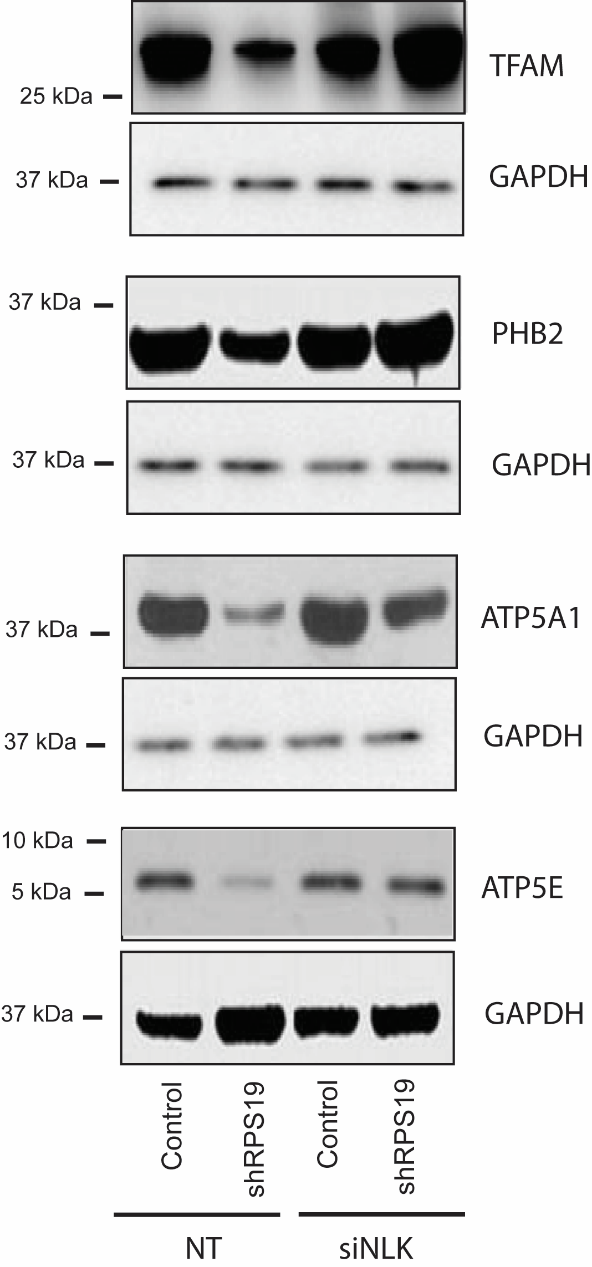
**

**Supplemental Figure 5**. Deregulated translation of mitochondrial biogenesis transcripts reduces protein expression through NLK. Cord Blood CD34+ progenitors transduced with shRNA against luciferase (Control) or shRNA against RPS19 (shRPS19) in conjunction with siRNA against NLK (siNLK) or a non-targeting sequence (NT). Cells were differentiated for 5 days and 4 identical replicates were pooled, prior to lysis. Lysates were probed for TFAM, ATG4, RPS6, GATA, p38 and NLK protein expression by western blot analysis. Accompanies Figure 3.


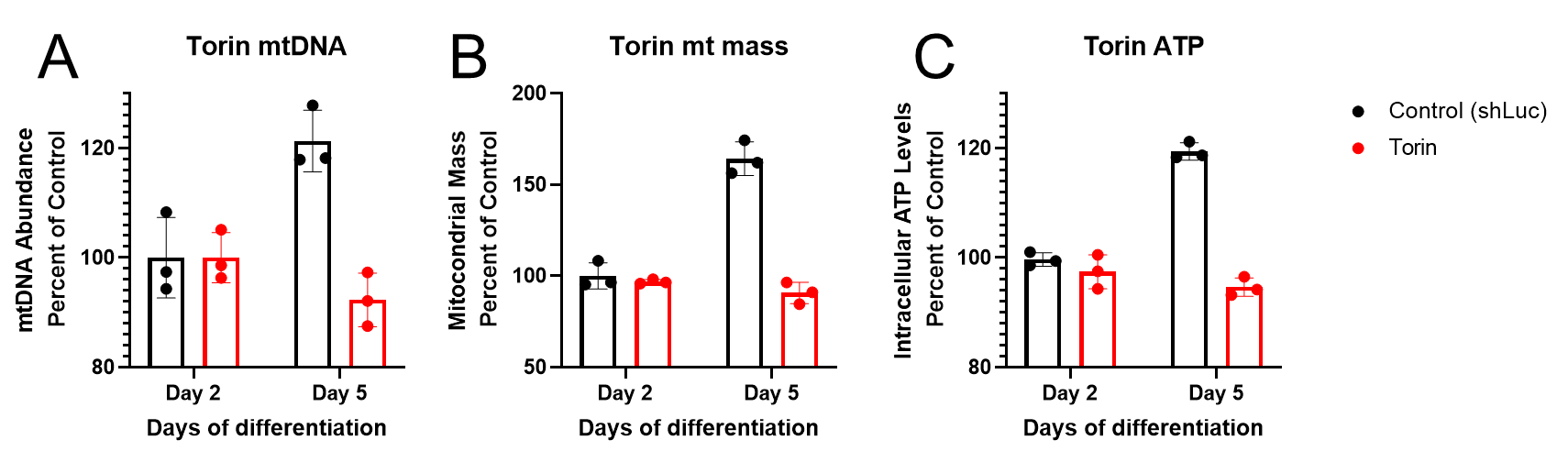


**Supplemental Figure 6**. mTORC1 is required for upregulation of mitochondrial biogenesis in erythropoiesis. Human cord blood CD34+ HSPCs transduced with control or shRNA against RPS19 were differentiated in the presence or absence of torin and assessed for mtDNA (A), mitochondrial mass (B) intracellular ATP (C). Three individual repeats were performed in triplicate. Data are represented as mean ± SD of the triplicate means and significance is defined as p<0.05 (n=3). Accompanies Figure 4.


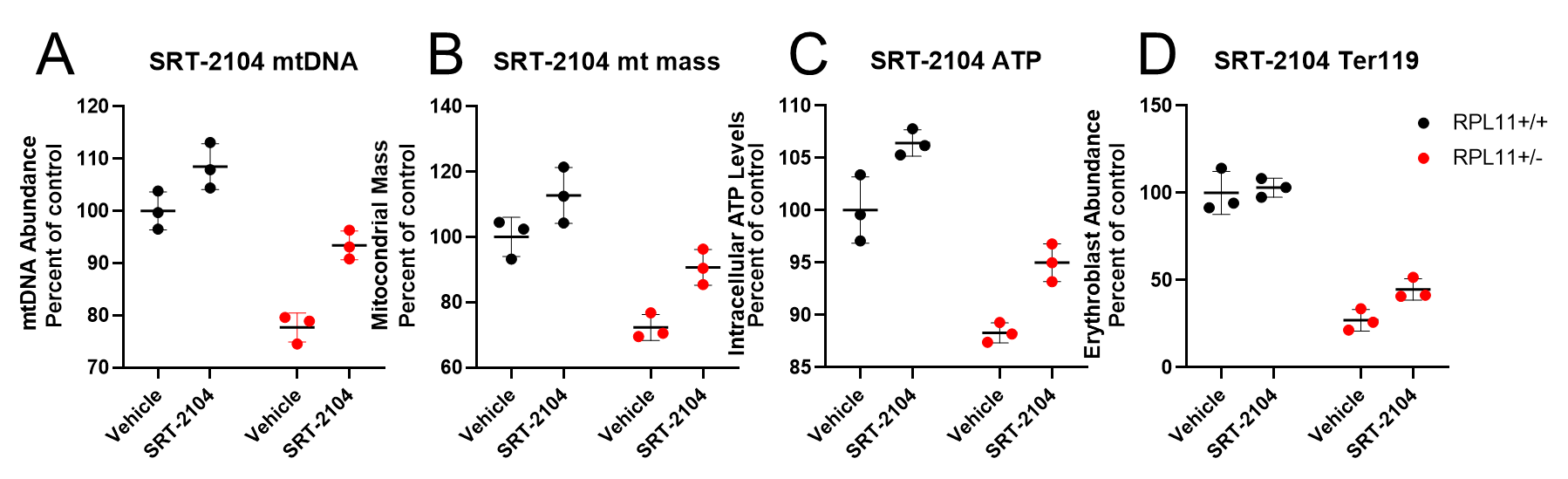


**Supplemental Figure 7**. Mitochondrial biogenesis inducer SRT-2104 improves erythropoiesis in ribosome insufficient mouse cells. Control and ribosome insufficient HSPCs were differentiated in the presence or absence of SRT-2104 and assessed for mtDNA (A), mitochondrial mass (B) intracellular ATP (C) and expansion of Ter119+ erythrocytes (D). Three individual repeats were performed in triplicate. Data are represented as mean ± SD of the triplicate means and significance is defined as p<0.05 (n=3). Accompanies Figure 4.


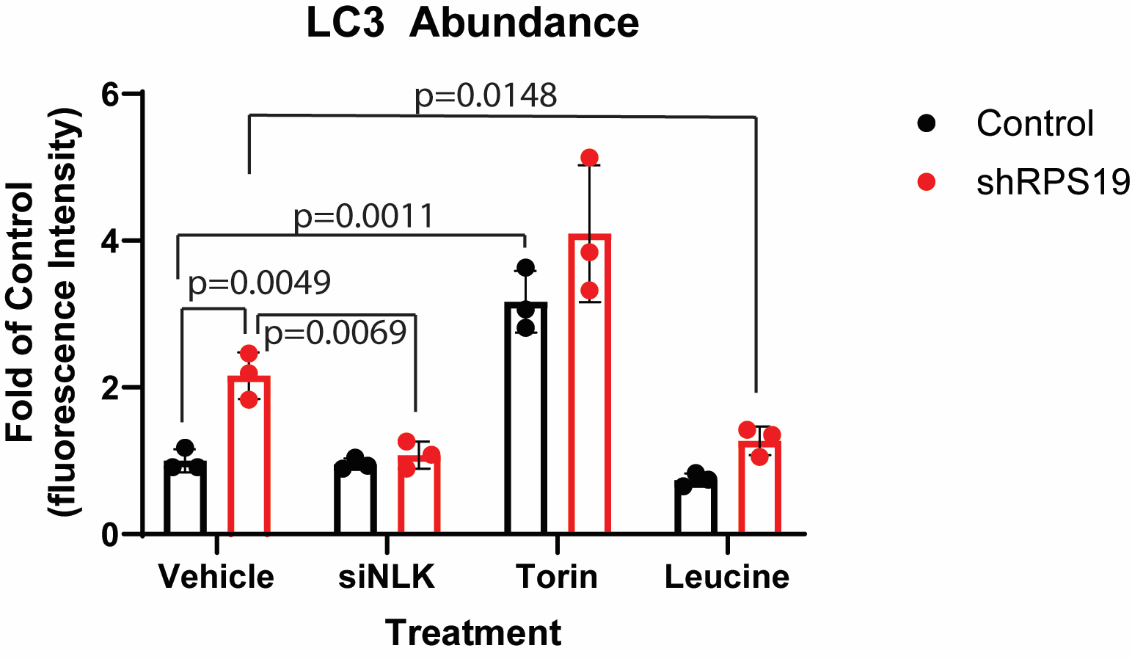


**Supplemental Figure 8**. NLK and mTORC1 activity influence autophagy in ribosome insufficient erythroid progenitors. Cord blood progenitors were transduced with shRNA against luciferase (black) or RPS19 (red) in conjunction with DMSO, torin, leucine or siRNA against NLK and differentiated for 7 days. CD71+ erythroid progenitors were sorted by FACS and incubated with an intracellular stain visualizing LC3-containing autophagosomes and absorbance measured at a wavelength of 520nm. Three individual repeats were performed in triplicate. Data are represented as mean ± SD of the triplicate means and significance is defined as p<0.05 (n=3). Accompanies Figure 5.

**Supplemental Table 1.** qRT-PCR Primer and shRNA sequences.

Human mtDNA.fwd CCCTAAAACCCGCCACATCT

Human mtDNA.rvs GAGCGATGGTGAGAGCTAAGGT

Mouse mtDNA.fwd CCCTAAAACCCGCCACATCT

Mouse mtDNA.rvs CCTTGACGGCTATGTTGATG

Human TFAM shRNA

CCGGGTAAGTTCTTACCTTCGATTTCTCGAGAAATCGAAGGTAAGAACTTACTTTTT

Human PHB2 shRNA CCGGGCGAGTGTTGTCTCGACCCAACTCGAGTTGGGTCGAGACAACACTCGCTTTTTG
